# A comparative analysis of unilateral biportal endoscopic and open laminectomy in multilevel lumbar stenosis

**DOI:** 10.3389/fneur.2024.1409088

**Published:** 2024-12-24

**Authors:** Jian-yuan Ouyang, Qi-Yuan Yang, Lan-lan Chen, Qin Li, Yu-hao Zheng, Xiao Luo, Bing Tan

**Affiliations:** ^1^Department of Spine Surgery, The Third Hospital of Mianyang, Sichuan Mental Health Center, Mianyang, China; ^2^Department of Neurology, The Third Hospital of Mianyang, Sichuan Mental Health Center, Mianyang, China

**Keywords:** multilevel lumbar spinal stenosis, unilateral biportal endoscopy, open lumbar decompression, spinal endoscopy, unilateral approach bilateral decompression

## Abstract

**Background:**

Approximately 103 million people across the globe suffer from symptomatic lumbar spinal stenosis, impacting their health and quality of life. The unilateral biportal endoscopic technique is effective for treating single-segment degenerative lumbar spinal stenosis and is seen as a viable alternative to traditional open lumbar laminectomy. However, research on the application of this technique for multilevel lumbar spinal stenosis remains lacking.

**Objective:**

To compare the clinical effects of unilateral biportal endoscopy (UBE) and open lumbar decompression (OLD) in the treatment of multilevel lumbar spinal stenosis (MLSS).

**Methods:**

This retrospective study was conducted from February 2019 to December 2023 and compared the outcomes of Multilevel UBE surgery to OLD. The included patients were divided into two groups, namely the UBE group (*n* = 42, 86 surgical segments) and the OLD group (*n* = 40, 82 surgical segments). At the 1-year follow-up, the imaging findings, visual analogue scale (VAS), Oswestry disability index (ODI), and Zurich Claudication Questionnaire (ZCQ) were assessed. MRI measurements of the dural sac (CSA) and paravertebral cross-sectional area (PMA) were taken before surgery and at the final follow-up.

**Results:**

The surgical segments of the two groups primarily consisted of adjacent segments (UBE 78.6% vs. OLD 78.8%), with a higher proportion of bilateral decompression in the OLD group (UBE 24.4% vs. OLD 28.0%). Preoperative imaging evaluation indicated a higher prevalence of grade C (severe stenosis) compared to grade D (severe stenosis) in both groups (UBE 74.4% vs. OLD 72%). The OLD group exhibited significantly greater blood loss compared to the UBE group (147.63 ± 26.55 vs. 46.19 ± 25.25 mL, *p* < 0.001). In addition, the duration of hospitalization in the OLD group was notably longer compared to the UBE group (7.58 ± 1.39 vs. 4.38 ± 1.56 days, *p* < 0.05). Paravertebral muscle atrophy (PMA) in the UBE group was significantly lower than in the OLD group (3.49 ± 3.03 vs. 5.58 ± 3.00, *p* < 0.05). Significantly elevated serum creatine kinase (CK) levels were observed in both groups, peaking at 1-day post-surgery, with the UBE group showing significantly lower levels than the OLD group (108.1 ± 12.2 vs. 364.13 ± 20.24 U/L, *p* < 0.05). On postoperative day 7, a significant decrease in liver enzyme levels was found in UBE group compared to the preoperative levels (61.81 ± 7.14 vs. 66.10 ± 8.26 U/L, *p* < 0.05). The Oswestry Disability Index (ODI) and Zurich Claudication Questionnaire (ZCQ) scores at 1 week, 6 months, and 1 year post-operation showed significant improvement compared to the preoperative scores in both groups (*p* < 0.05). The study found statistically significant differences in both the Visual Analog Scale (VAS) score (2.28 ± 0.59 vs. 2.85 ± 0.74, *p* < 0.05) and the Oswestry Disability Index (ODI) score (36.28 ± 2.03 vs. 37.57 ± 1.98, *p* < 0.05) at 1 week post-surgery between the two groups. However, no significant variations in scores were noted between preoperative and postoperative time points at other follow-up intervals.

**Conclusion:**

The unilateral biportal endoscopic technique was applied to treat multilevel lumbar spinal stenosis, demonstrating decreased intraoperative bleeding and lower postoperative muscle-related complications compared to open lumbar decompression. Furthermore, UBE was found to promote early mobilization.

## 1 Introduction

Symptomatic lumbar spinal stenosis, characterized by symptoms such as low back pain, leg radiation pain, and intermittent claudication, affects approximately 103 million individuals globally, significantly impacting their health and quality of life (1, 2). Multi-segmental lumbar spinal stenosis may be present in some cases, and the surgical approach for patients with symptomatic lumbar spinal stenosis is generally determined by considering the patient’s symptoms, signs, and imaging results (3). Recent advances in spinal endoscopy have sparked a resurgence of interest in minimally invasive treatment for lumbar spinal stenosis. Specifically, UBE allows a minimally invasive and precise approach to managing multi-segmental lumbar spinal stenosis (4, 5).

The predominant surgical approach for addressing degenerative lumbar spinal canal stenosis (DLSS) is lumbar decompression with or without fusion (6). Prior research has demonstrated comparable clinical outcomes and complication rates between fusion and non-fusion surgeries for lumbar spinal stenosis. However, fusion surgery typically requires a longer hospitalization period (7). Previous reports have also suggested that non-fusion surgery may mitigate excessive alterations in adjacent segment kinematics, thereby presenting a lower risk of adjacent segment disorder (8). Research indicates that open decompressive laminectomy, a traditional surgical approach, significantly enhances Oswestry disability index scores and quality of life in individuals with lumbar spinal stenosis (9). Nevertheless, the extensive muscle stripping and structural damage to the articular process associated with open surgery can compromise long-term surgical outcomes (10). Furthermore, surgical methods are constantly being optimized to minimize trauma, muscle damage, and incisional damage.

In recent years, the unilateral approach bilateral decompression (UBE) technique has been recognized for its ability to minimize tissue damage and achieve successful outcomes in the treatment of degenerative lumbar spinal stenosis (11). Existing research has demonstrated the efficacy of UBE in improving postoperative functional outcomes and clinical scores for single-segment lumbar spinal stenosis (12–14), but studies on the application of UBE in treating multilevel stenosis are lacking. In this study, a retrospective analysis was conducted on 82 patients who underwent treatment at our hospital between 2019 and 2023 to investigate the safety and efficacy of unilateral biportal endoscopic (UBE) surgery in the treatment of multi-segmental lumbar spinal stenosis. Patients were categorized into two groups based on the surgical method employed - the open lumbar decompression (OLD) group and the UBE group - and their initial clinical outcomes were assessed.

## 2 Materials and methods

### 2.1 Patient group

In this retrospective study, a total of 82 patients who underwent lumbar discectomy surgery were divided into two groups. The unilateral biportal endoscopic (UBE) group comprised 42 patients who underwent 86 vertebral-level surgeries, and the conventional open lumbar decompression (OLD) group included 40 patients who underwent 82 vertebral-level surgeries. The demographic and clinical characteristics are presented in Table 1. Before treatment, all patients provided written informed consent, and the study was approved by the Medical Ethics Committee and Institutional Review Board (Approval No. 178, 2023).

**Table 1:** Demographic information and follow-up.

Variables	UBE (*N* = 42)	OLD (*N* = 40)	*p*-value
Age (years)	63.9 ± 14.2	66.0 ± 13.6	0.506
Sex (%)			
Female	24 (59.5%)	22 (60.0%)	0.793
Male	18 (40.5%)	18 (40.0%)	
BMI (kg/m^2^)	27.5 ± 2.4	27.2 ± 1.9	0.432
Operative level, *n* (%)			
Non-adjacent segments	6 (14.3%)	7 (17.5%)	0.690
Adjacent segments	36 (85.7%)	33 (82.5)	
Schizas classification, *n* (%)			
C	64 (74.4%)	69 (72.0%)	0.718
D	22 (25.6%)	23 (28.0%)	
Pressure reduction mode, *n* (%)			0.396
Unilateral decompression	62 (75.6%)	60 (69.8%)	
Bilateral decompression	20 (24.4%)	26 (30.2%)	
Duration of disease (days)	37.2 ± 8.2	35.8 ± 9.6	0.478
Comorbidity			
Hypertension	26 (55.0%)	22 (61.9%)	0.551
Cardiopathy	20 (50%)	16 (31.8%)	
Lung disease	14 (51.9%)	13 (48.1%)	
DM	23 (54.8%)	19 (47.5%)	
Follow-up (months)	15.3 ± 2.5	16.1 ± 2.1	0.065

Values are presented as mean ± standard deviation unless otherwise indicated. BMI, body mass index; DM, diabetes mellitus.

The study’s inclusion criteria were: (1) MRI and CT examinations revealing spinal stenosis in at least two segments of the lumbar spine with a Schizas grade of ≥ C; (2) Diagnosis of lumbar spinal stenosis (LSS) with clinical symptoms and signs corresponding to the imaging findings; (3) Presence of intermittent claudication, varying degrees of low back pain or lower limb pain, and numbness; and (4) Modified Pfirrmann grade of the lesion segment not exceeding 7; (5) Six months of conservative treatment proved to be ineffective, with the condition severely impacting both work and daily life.

The exclusion criteria were lumbar spondylolisthesis grade ≥ II degree or lumbar instability, obvious kyphosis or scoliosis deformity, history of posterior decompression of the lesion segment, intervertebral disc-related diseases (e.g., discitis, intervertebral space infection, tumor, spinal tuberculosis), severe medical conditions that precluded surgery, and mental health issues that could affect functional evaluation. Additionally, patients with a follow-up time of less than 1 year were not included in the study.

## 3 Operative method

### 3.1 Unilateral biportal endoscopy (UBE)

The patient was placed in a prone position under general anesthesia. The surgical site was maintained in the horizontal plane and the abdomen was suspended, with a slight flexion of the lumbar bridge. Standard disinfection procedures were followed, including the application of a skincare membrane, placement of drapes, connection of endoscopy equipment, utilization of a radiofrequency electrode knife, grinding drill, and perfusion system. C-arm X-ray fluoroscopy was employed for precise positioning of the intervertebral space. The working channel and observation channel were positioned in their respective locations. 3,000 mL of normal saline was utilized as the flushing solution, positioned at a height of 50–60 cm above the surgical incision plane.

Unilateral decompression was done for patients with mild unilateral lower limb symptoms and minor central spinal canal or nerve root canal stenosis. Superficial soft tissue and part of the ligamentum flavum were cleared, preserving its deeper part. The lamina and ligamentum flavum were gradually removed using a bone knife, spatula, and lamina bite forceps, from the upper lamina’s head to the midline of the pedicle. The ipsilateral walking root was decompressed to reveal the inner edge and midline of the ipsilateral pedicle. The nerve root’s shoulder and axilla were fully exposed, and the distal end of the nerve root canal was examined.

Bilateral decompression was conducted for severe central spinal canal or double nerve root canal stenosis causing lower limb symptoms. Post decompression on one side, the deep ligamentum flavum was temporarily retained to aid the other side’s operation. The midline of the spinal canal was identified using the middle fissure of the cephalic deep ligamentum flavum. The contralateral vertebral plate and ventral bone of the inferior articular process were removed along the base of the cephalic spinous process, near the deep layer of the ligamentum flavum. The superficial layer of the ligamentum flavum was also removed. This exposed the medial edge of the superior articular process, allowing decompression of the contralateral lateral recess using tools like bone-biting forceps and curettes. The resection was extended to the contralateral pedicle’s medial wall and mid-pedicle level. A probe checked for nerve root canal narrowing and assessed the need for outlet root decompression. Post-decompression, the contralateral disc was examined and treated. After successful decompression, each nerve root was re-examined, bleeding was controlled, a drainage tube was placed endoscopically, the area was disinfected, the incision sutured, and a sterile dressing applied.

### 3.2 Open lumbar decompression (OLD)

During fluoroscopy, the posterior median incision was used to dissect the paravertebral muscles. The upper and lower edges of the adjacent lamina were incised along the medial part of the articular process, and the lateral semi-articular process was removed. The thickened ligamentum flavum was removed to completely decompress the nerve root and dura mater. The decompression method was also determined according to the patient ‘s symptoms and imaging results. In cases where contralateral decompression was deemed necessary, decompression of the contralateral recess was conducted from the base of the spinous process until adequate decompression of the contralateral nerve root was attained. Similar procedures were performed on the other surgical segments. Following the achievement of sufficient hemostasis, a drainage tube was inserted, and the incision was subsequently sutured.

### 3.3 Evaluation

#### 3.3.1 Demographic and perioperative data collection

Patient demographics and perioperative data were analyzed by reviewing the medical records. Demographic information was collected, including age, gender, body mass index (BMI), smoking status, duration of symptoms, length of hospital stay, follow-up period, and preoperative diagnosis. Perioperative variables such as surgical segments, operative time, estimated blood loss, and intraoperative and postoperative complications within 1 year post-surgery were also documented. Creatine kinase levels were measured preoperatively, as well as on day 1 and day 7 postoperatively, as outlined in Table 2.

**Table 2:** Comparison of intraoperative data between the two operations.

Variables	UBE (*N* = 42)	OLD (*N* = 40)	*P*-value
Operative time (min)	151.67 ± 9.82	93.42 ± 5.02	<0.001**
EBL (ml)	46.19 ± 25.25	147.63 ± 26.55	<0.001**
Length of hospital stay (days)	4.38 ± 1.56	7.58 ± 1.39	<0.001**
Creatine Kinase (U/L)			
Preop	61.38 ± 7.14	61.3 ± 8.48	0.521
Postoperative day 1	108.1 ± 12.17	364.13 ± 20.24	<0.001**
Postoperative day 7	61.81 ± 7.14	66.10 ± 8,26	0.014*
Perioperative complications, *n*(%)	2	5	0.235
Dural sac tearing	2 (4.8%)	3 (7.5%)	
Incision infection	0	2 (5%)	

Values are presented as mean ± standard deviation. EBL, estimated blood loss. *p < 0.05, **p < 0.001.

#### 3.3.2 Clinical and radiographic results

The study evaluated clinical and imaging outcomes of patients with lumbar spinal stenosis (MLSS) using the Visual Analogue Scale (VAS), Oswestry Disability Index (ODI), and Zurich Claudication Questionnaire (ZCQ) to assess subjective symptoms and objective clinical signs. Preoperative and postoperative VAS, ODI, and ZCQ scores were recorded at 1 week, 6 months, and 12 months postoperatively. Additionally, the cross-sectional area (CSA) of the spinal canal and the paraspinal muscle area (PMA) were measured preoperatively, postoperatively, and at the last follow-up using the hospital’s Picture Archiving and Communication System (PACS).

### 3.4 Statistical analysis

Statistical analysis was conducted in accordance with standard procedures. Data were reported as mean ± standard deviation unless otherwise specified. Radiological evaluations were performed by a board-certified spine surgeon. Interobserver reliability was assessed using intraclass correlation, and the results were classified as poor (0–0.39), moderate (0.4–0.74), or excellent (0.75–1). The t-test and paired t-test were utilized to compare continuous variables and assess differences within and between groups, respectively. Chi-square analysis was employed for categorical variables in the presence of variations. In this study, statistical significance was set at *p* < 0.05. All statistical analyses were performed using SPSS software (SPSS Inc., Chicago, IL, USA, version 25.0).

## 4 Results

### 4.1 Demographic results

The study comprised 82 patients, including 42 in the UBE (unilateral biportal endoscopy) group and 40 in the OLD group. Table 1 presents the demographic and baseline characteristics of these groups. The baseline demographic data (Table 1) indicated no significant differences between the two groups. The majority of patients in both groups exhibited involvement of adjacent segments (UBE 78.6%, OLD 78.8%), and bilateral decompression was the preferred method in both groups (UBE 24.4%, OLD 28.0%). Preoperative imaging assessment showed a greater prevalence of Grade C (severe stenosis) compared to Grade D (more severe stenosis) (UBE 74.4%, OLD 72%).

### 4.2 Surgical outcomes and complications

A comparison of surgical-related data between the UBE and OLD groups revealed that the OLD group had a significantly higher volume of blood loss (147.63 ± 26.55 vs. 46.19 ± 25.25 mL) (*p* < 0.001; Table 2) and a longer length of hospital stay (7.58 ± 1.39 vs. 4.38 ± 1.56 days, *p* < 0.001, Table 2) compared to the UBE group. In contrast, no significant differences in perioperative complications were found between the two groups, with dural tears occurring in 2 cases in the UBE group and 3 cases in the OLD group, along with 2 cases of incision infection in the OLD group.

### 4.3 Clinical results

These complications resolved within a month in both groups. Furthermore, the change in dural sac cross-sectional area (CSA) showed no significant difference between the UBE and OLD groups (UBE: 0.69 ± 0.15 cm, OLD: 0.73 ± 0.16, *p* > 0.05, Table 2). Significantly elevated serum creatine kinase (CK) levels were observed in both groups, peaking on postoperative day 1. Still, the UBE group exhibited significantly lower levels compared to the OLD group (108.1 ± 12.2 vs. 364.13 ± 20.24 U/L, *p* < 0.05, Table 2). Furthermore, when comparing the UBE group to the OLD group, it was observed that the UBE group exhibited lower levels of CK (61.81 ± 7.14 vs. 66.10 ± 8.26 U/L, *s* < 0.05, as shown in Table 2). The Oswestry Disability Index (ODI) and Zurich Claudication Questionnaire (ZCQ) scores at 1 week, 6 months, and 1 year post-operation showed significant improvement compared to the preoperative scores in both groups (*p* < 0.05, Table 3). The study found statistically significant differences in both the Visual Analog Scale (VAS) score (2.28 ± 0.59 vs. 2.85 ± 0.74, *p* < 0.05) and the Oswestry Disability Index (ODI) score (36.28 ± 2.03 vs. 37.57 ± 1.98, *p* < 0.05) at 1 week post-surgery between the two groups. However, no significant variations in scores were noted between preoperative and postoperative time points at other follow-up intervals. The change in CSA post-surgery showed no statistical significance between the two groups (0.69 ± 0.15 vs. 0.73 ± 0.16, *p* > 0.05). However, the UBE group induced significantly lower paraspinal muscle atrophy compared to the OLD group (3.49 ± 3.03 vs. 5.58 ± 3.00, *p* < 0.05, Table 3).

**Table 3:** Comparison of clinical and imaging data after two kinds of operation.

Scoring system	UBE (*N* = 42)	OLD (*N* = 40)	*P*-value
VAS			
Preop (mean score)	5.59 ± 0.50	5.68 ± 0.62	0.591
Postop (1 week)	2.28 ± 0.59	2.85 ± 0.74	0.013*
Follow-up at 6 months	1.85 ± 0.35	1.70 ± 0.46	0.088
Follow-up at 1 years	1.73 ± 0.45	1.62 ± 0.49	0.271
*p*-value (pre vs.post)	<0.001**	<0.001**	
ODI			
Preop (mean score)	61.79 ± 3.31	62.33 ± 3.67	0.488
Postop (1 week)	36.28 ± 2.03	37.57 ± 1.98	0.005*
Follow-up at 6 months	22.73 ± 3.60	22.98 ± 3.57	0.099
Follow-up at 1 years	19.79 ± 2.80	19.65 ± 2.47	0.204
p-value (pre vs.post)	<0.001**	<0.001**	
ZCQ			
Preop (mean score)	66.00 ± 3.28	66.25 ± 3.47	0.783
Postop (1 week)	38.83 ± 4.38	39.08 ± 4.32	0.802
Follow-up at 6 months	22.74 ± 3.60	22.97 ± 3.57	0.766
Follow-up at 1 years	19.79 ± 2.79	19.65 ± 2.47	0.817
p-value (pre vs.post)	<0.001**	<0.001**	
The changes of CSA before and after operation	0.69 ± 0.15	0.73 ± 0.16	0.096
The changes of PMA before and after operation	3.49 ± 3.03	5.58 ± 3.00	<0.001**

Values are presented as mean ± standard deviation. VAS, visual analogue scale; ODI, Oswestry Disability Index; ZCQ, Zurich Claudication Questionnaire; CSA, cross-sectional area; PMA, paravertebral Muscle area. *p < 0.05, **p < 0.001.

### 4.4 Typical case

**Figure 1:**
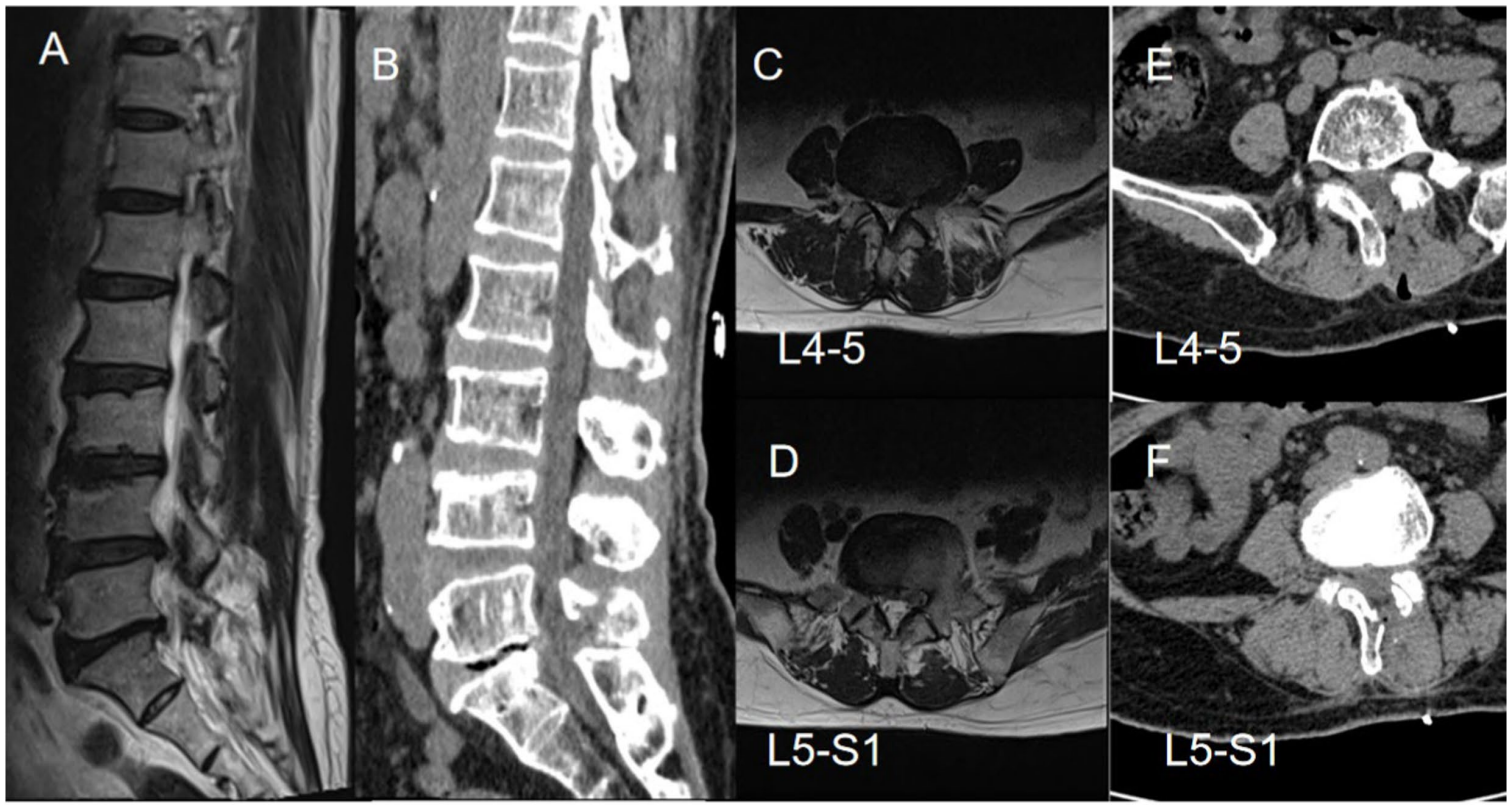
A 70-year-old female patient with disc herniation at the L4-5 and L5-S1 levels presented with pain and numbness in the lateral and posterior regions of the left lower leg, along with pain in the lateral aspect of the right calf. Preoperative axial and sagittal MR images revealed significant spinal canal stenosis at the L4-5 and L5-S1 levels **(A–D)**. Subsequent postoperative three-dimensional CT scans displayed an increase in the width of the spinal canal following the surgical intervention **(E,F)**.

**Figure 2:**
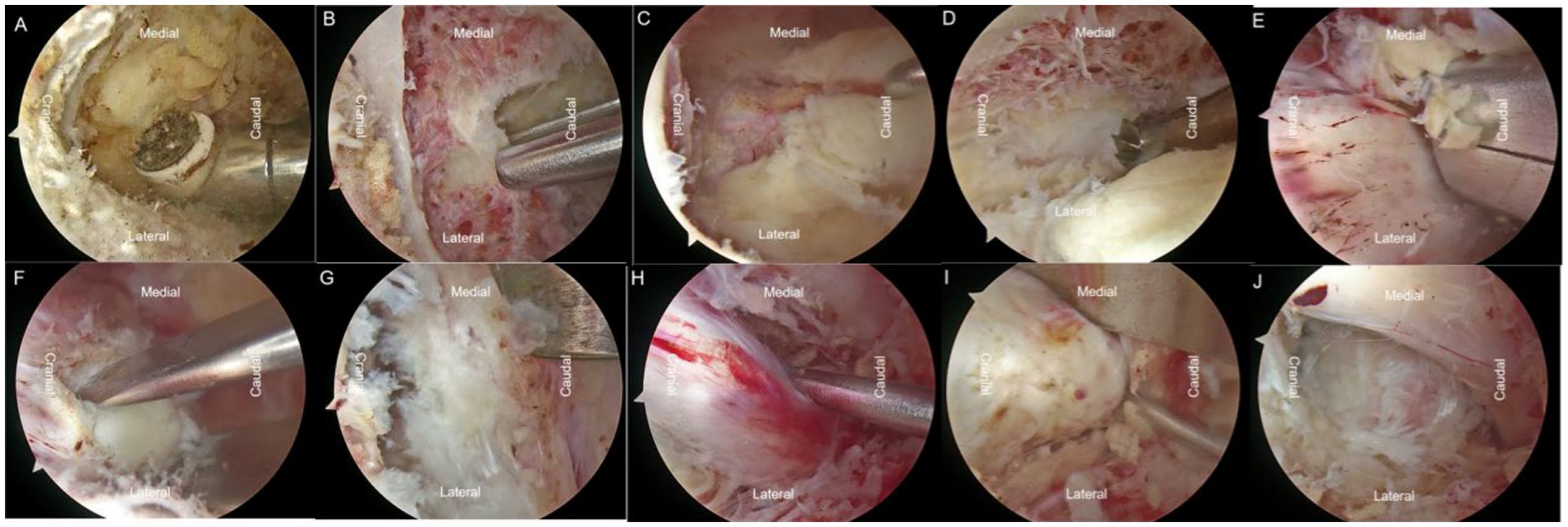
Based on the patient’s condition, L4-5 ULBD (unilateral laminotomy for bilateral decompression) and L5-S1 UBE (unilateral biportal endoscopy) surgeries were performed. **(A)** The radiofrequency electrode was used to create a pathway through the interlaminar muscles. **(B)** Laminar rongeurs and high-speed burrs were utilized to remove the laminar tissue on the left side. **(C)** The ligamentum flavum was preserved to facilitate further operations. **(D–E)** Removal of the base of the spinous process, crossing the V-point, was performed for over-the-top decompression, effectively decompressing the contralateral recess. Contralateral disc material was excised. **(F–H)** Decompression on the same side and excision of ipsilateral disc material were performed. **(I)** Disc material was removed on the left side at L5-S1. **(H)** Exploration and loosening of the nerve root were conducted.

**Figure 3:**
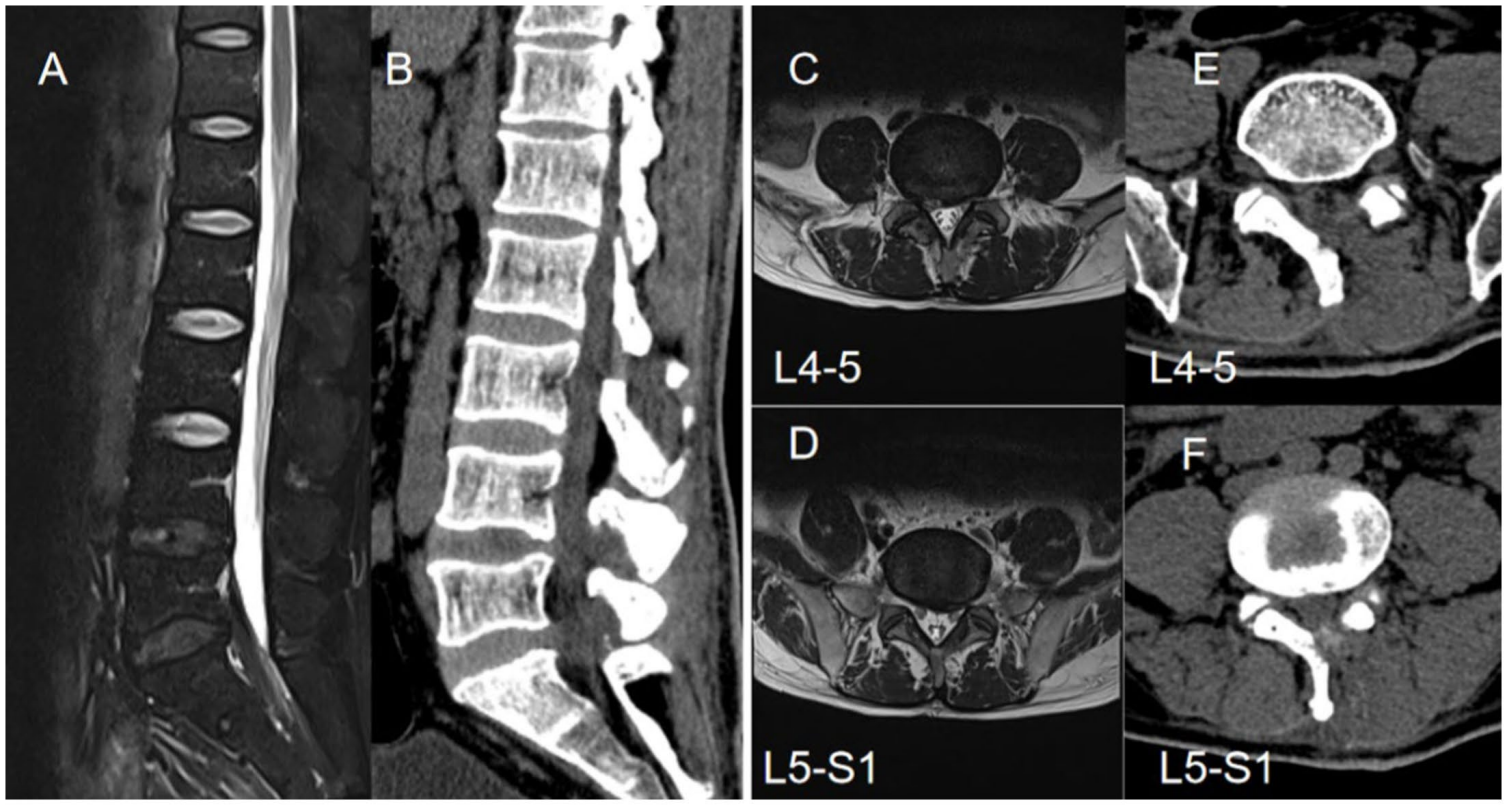
A 49-year-old male patient presented with L4-5 disc herniation and L5-S1 epidural lipomatosis, exhibiting clinical symptoms of pain and numbness in both lower limbs, with more severe manifestations on the right side **(A,B)**. Preoperative axial and sagittal magnetic resonance imaging revealed L4-5 disc herniation and increased L5-S1 epidural fat, leading to spinal stenosis**(C,D)**. Subsequent axial CT findings post-unilateral laminectomy demonstrated postoperative spinal canal widening on three-dimensional CT scan **(E,F)**.

The typical images are shown in Figures 1–3.

## 5 Discussion

The prevalence of multilevel lumbar spinal canal stenosis has increased in recent years due to the aging population and the advancements in imaging technology. Nevertheless, not all affected segments necessitate surgical intervention, and determining which segments should be operated on should be based on the patient’s clinical data (15). The accurate selection of the appropriate surgical approach has emerged as a topic of debate among surgeons. Research indicates that around 30% of patients who undergo traditional open decompression surgery may necessitate revision surgery or spinal fusion, primarily due to postoperative spinal instability (16). Furthermore, advanced minimally invasive techniques, such as endoscopic decompression and selective segmental decompression, have been developed to provide effective treatment options for multilevel lumbar spinal canal stenosis (17). In such cases, the patient’s symptoms and signs may be examined to identify segments with severe lesions or prominent symptoms for decompression, thereby mitigating iatrogenic spinal instability and enhancing clinical outcomes (18). The unilateral biportal endoscopy (UBE) technique not only replicates the outcomes of open surgery but also offers the benefits of decreased trauma, reduced blood loss, and notably shorter hospitalization periods (19). The procedural and cognitive framework of UBE closely resembles that of open surgery, which substantially decreases the learning curve (20). Several publications have documented the positive outcomes of utilizing the UBE technique in the treatment of single-segment lumbar spinal stenosis (11, 12, 14, 21, 22). However, reports investigating the clinical efficacy of UBE in multilevel lumbar spinal stenosis remain scarce.

Kim et al. (21) conducted a multicenter retrospective study involving 60 patients who underwent single-segment UBE surgery and 81 patients who underwent open surgery. Their results revealed comparable clinical outcomes in terms of pain management, functional disability, and patient satisfaction between the two groups. Our results are in accordance with these findings, indicating that the UBE technique can be applied to multilevel procedures. On the other hand, Tan et al. ^14^not only examined historical clinical data but also assessed the spinal canal area before and after surgery. The outcomes revealed a significant increase in postoperative CSA in both cohorts, affirming the beneficial decompressive impact of UBE from a radiological standpoint. Our study conducted a statistical analysis of the cross-sectional area (CSA) before and after surgery for each surgical segment, revealing no statistically significant difference between the two groups. Overall, both postoperative symptoms and imaging assessments suggest that the use of the UBE technique in the treatment of multilevel lumbar spinal stenosis can yield comparable postoperative efficacy to traditional open surgery.

In addition to demonstrating comparable surgical efficacy to open surgery, UBE offers several advantages in the treatment of multilevel lumbar spinal stenosis.

UBE surgery has been shown to lead to decreased trauma and muscle dissection, as indicated by a reduction in blood loss. A recent meta-analysis of nine studies involving 823 patients with single LSS segments demonstrated that UBE surgery resulted in significantly lower blood loss compared to traditional open surgery (mean difference = −6.14, 95% confidence interval: −9.32 to −2.96, *p* = 0.0002) (17). Our study further corroborates these findings, supporting the notion that UBE surgery is associated with reduced blood loss. The measurement of early postoperative serum creatine kinase (CK) levels serves as a valuable tool in assessing the degree of muscle damage following spine surgery (23).In our study, it was observed that the early postoperative serum creatine kinase (CK) levels in the UBE group were significantly lower compared to those in the OLD group, thus confirming the hypothesis of minimal tissue damage. Furthermore, alterations in postoperative paraspinal muscle area (PMA) provide a more direct and accurate assessment of muscle damage (14).Surgical interventions for multilevel lumbar spinal stenosis typically entail a single incision, which may inadvertently expose unnecessary muscle tissue. Employing the UBE technique enables precise decompression of specific surgical segments without the requirement to expose muscles in non-operative regions, thereby reducing surgical trauma. A study has observed changes in the paraspinal muscle area (PMA) post-surgery for single-segment lumbar spinal stenosis, indicating a notably reduced degree of paraspinal muscle atrophy in the UBE group (4.50 ± 0.60) compared to the OLD group (11.42 ± 0.87). Similarly, the current study evaluated alterations in PMA, demonstrating a significantly lower level in the UBE group compared to the open surgery group. These results support the occurrence of minimal paraspinal muscle damage following UBE surgery as evidenced by imaging analysis.

The unilateral approach for bilateral decompression (ULBD) using unilateral biportal endoscopy (UBE) is a recommended method for addressing severe lumbar spinal canal stenosis (24). ULBD involves over-the-top decompression while preserving facet joints, neural arch, and musculature on the contralateral side, and has been shown to effectively reduce postoperative instability and minimize damage to spinal structures and muscles (25, 26). Applying the unilateral approach in bilateral decompression endoscopic surgery enables a thorough and precise visualization of the contralateral lateral recess structure by adjusting the endoscopic view. This facilitates a complete and safe direct visual decompression of the neural structures, leading to a significant reduction in symptoms and disability, increased acceptance of reoperation rates, and enhancement of health-related quality of life (19, 27). In contrast, bilateral decompression open surgery, typically involving bilateral laminectomy, involves greater tissue damage due to the absence of an endoscopic system, rendering precise decompression challenging, increasing trauma, and substantially elevating the likelihood of nerve damage. Conversely, the unilateral bilateral decompression approach utilizing the UBE method facilitates direct visualization during decompression, enhancing hemostasis and reducing the risk of surgical nerve damage. Our research indicates an incidence of dural sac injury of 4.8% with UBE, compared to 7.5% with open laminectomy.

The separation of observation and surgical channels in UBE is significant as it offers benefits such as surgical flexibility, a clear surgical field, and bilateral decompression (12). Additionally, it enables the utilization of a variety of surgical instruments for spinal canal decompression. UBE is employed not only in the treatment of spinal canal stenosis and lumbar disc herniation but also in addressing other intraspinal pathologies. Research conducted by Kim SK and colleagues has demonstrated the application of UBE in the management of lumbar spine tumors, utilizing advanced imaging technology and a variety of surgical tools for decompressing the spinal canal and safely removing tumors, resulting in successful outcomes (28). Wang et al. (29) similarly found positive results in managing intraspinal extradural benign tumor lesions with UBE. In our study, UBE was performed on a patient with epidural lipomatosis (Case 2), who presented with spinal canal stenosis due to an enlarged epidural fat layer. The endoscopic view provided a clear visualization of the epidural fat structure, facilitating effective decompression.

One additional benefit of undergoing UBE surgery is the reduced length of hospitalization for patients. Our research findings indicate that the average hospital stay for multilevel UBE surgery was 4.38 ± 1.56 days, which was notably shorter than that for open discectomy performed under comparable circumstances (7.58 ± 1.39 days). On the other hand, single-level surgery showed no significant difference in hospital stay duration, suggesting that multilevel UBE surgery may not significantly prolong the patient’s hospitalization. However, UBE surgery is associated with longer operative times, which is primarily attributed to extended preoperative preparations.

This study is subject to certain shortcomings and limitations. Primarily, it is a retrospective analysis, which inherently involves considerations such as patient acceptance, economic status, individual differences, and physician preferences in the preoperative selection of surgical interventions. However, the absence of preoperative blinding or randomization has potentially compromised the accuracy and reliability of the findings. To further elucidate the relationship between the two surgical approaches, additional multi-center, prospective, randomized studies are warranted for confirmation.

## 6 Conclusion

UBE technology has been shown to effectively decrease intraoperative bleeding by enhancing the precision and accuracy of endoscopic-assisted minimally invasive surgery, minimizing damage to surrounding tissue. Additionally, UBE technology has been found to facilitate early ambulation post-surgery, leading to improved rehabilitation outcomes and overall quality of life for patients. Additionally, in cases of multi-segmental stenosis, a thorough assessment of preoperative imaging and symptoms is imperative in order to develop individualized treatment strategies.

## Data Availability

The original contributions presented in the study are included in the article/supplementary material, further inquiries can be directed to the corresponding author.
